# Creation of a new *Drosophila melanogaster* X chromosome balancer, *First Multiple 8* ( *FM8* ), using the CRISPR/Cas9 gene-editing system

**DOI:** 10.17912/micropub.biology.000663

**Published:** 2022-11-10

**Authors:** Savannah Muron, Steve Kucera, Brian Oliver, Leif Benner

**Affiliations:** 1 Section of Developmental Genomics, Laboratory of Biochemistry and Genetics, National Institute of Diabetes and Digestive and Kidney Diseases, National Institutes of Health, Bethesda, MD, USA; 2 Department of Biology, The University of Tampa, Tampa, FL, USA; 3 Department of Biology, Johns Hopkins University, Baltimore, MD, USA

## Abstract

Balancer chromosomes contain multiple inversions that work to suppress crossing over and prevent recovery of most recombinant chromosomes, allowing for alleles to be kept long-term without selection. These balancers are incredible tools, but some alleles within larger inverted segments are still lost to rare double crossover events between the balanced and balancer chromosomes. This study details a new methodology of producing balancer chromosomes using CRISPR/Cas9 gene editing technology to create new inversions within the largest segment of the common X chromosome balancer, *FM7c.* We were able to create a new X chromosome balancer, *FM8,* and anticipate that this process can be used to not only create other balancers in *Drosophila melanogaster,* but other model organisms as well.

**Figure 1. Cartoon of wildtype, FM7c , and FM8 chromosomes:**
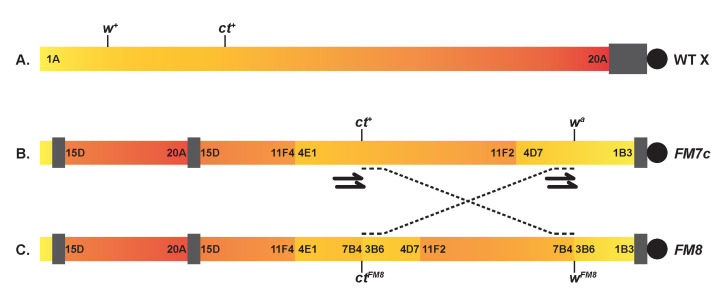
A) Wildtype X chromosome orientation in *Drosophila* . Gradient (yellow to red) represents the left (telomeric) to right (centromeric heterochromatin, filled bar and centromere, filled circle). The cytological positions 1-20 and location of *w* and *ct* are also indicated. B) *FM7c* X chromosome balancer. Each block represents the inverted segments at the marked cytological regions using the wildtype chromosome color gradient. C) Orientation of the new *FM8* X chromosome balancer created in this study. Arrows represent the location of the gRNAs used to create the new inversion. Dashed lines illustrate the new inversion created from the *FM7c* parent chromosome.

## Description

Balanced lethal alleles at two separate but linked loci and inversions that prevent transmission of recombined chromosomes enforce heterozygosity, such that parents and offspring are genetically identical (Muller 1918). Modern balancer chromosomes in *Drosophila* are essential genetic tools used for the maintenance of the allelic content of whole chromosomes that would be lost through the biological process of recombination. Additionally, marked balancers allows researchers to follow alleles and chromosomes through often complex multi-generational crosses (Miller et al. 2019). Balancer chromosomes contain nested inversions along their length that work to suppress crossing over and prevent the recovery of recombinant chromosomes in the next generation (most recombination events result in acentric and dicentric chromosomes), thus faithfully passing down the original chromosomal genotype from parents to offspring. However, balancers are not perfect, and exchange of alleles between the balancer and balanced chromosomes does occur by rare double crossover events between the non-inverted balanced chromosome and a non-inverted segment of a balancer (Miller et al. 2019; Miller, et al. 2020). Breakdowns in balanced stocks result in a loss of valuable genetic tools and research time.

One important example of a widely used balancer with a long non-inverted segment is the X chromosome balancer *First Multiple 7 (FM7,* Merriam 1968), where a female sterile mutation of *singed,* *sn ^X2^ ,* found on the *FM7c* chromosome, has been lost multiple times due to double crossover events within the 4E1-11F2 inversion (Miller et al. 2016a). We worked on several female sterile and female lethal genes in this region (e.g. *ovo* , *snf* , *Sxl* , *otu* ; Grammates et al. 2022) and wanted better balancing. Because the alleles of these genes we use are viable and fertile in males, we wanted hemizygous, and homozygous lethality in the balancer. To build a better balancer, we utilized a CRISPR/Cas9 genome editing system (Ren et al. 2013; Port and Bullock 2016; Benner and Oliver 2018) targeting this large problematic inversion of *FM7c* (4E1-11F2, Figure 1B). A new inversion in this segment would better suppress double crossover events within this region. In order to purposefully engineer a new inversion, we wanted to create a breakpoint within the 4E1-11F2 segment and another region outside this segment on *FM7c* . We decided to make an inversion at *cut* ( *ct* , within 4E1-11F2, Figure 1B), which is an essential gene but has viable alleles, and *white ^a^* ( *w ^a^* , within 4D7-1B3, Figure 1B), in the *FM7c* balancer chromosome. To achieve this objective, we created a polycistronic CRISPR gRNA construct (Port and Bullock 2016; Benner and Oliver 2018) that contained two gRNAs targeting the first intron of *w ^a^* (Grammates et al. 2022) and two gRNAs that targeted a region in between *ct* and the *ct ^6^* enhancer (Jack, 1985). We anticipated that deletion near the two cut sites would be common. The scheme we used allowed us to phenotypically screen for the presence of a new inversion independent of creating double mutants at both target loci. This is because small indels in the intronic and intergenic regions at either location should not disrupt either *w ^a^* or *ct* . However, the creation of an inversion separates the first exon of *w ^a^* from the rest of its reading frame and simultaneously separates the *ct ^6^* enhancer from being able to interact with the *ct* locus. We then took *FM7c* males containing both the polycistronic gRNA construct and Cas9 protein expressed in the germline (Ren et al. 2013) and crossed them to *y ^1^ w ^1^ ct ^6^* females.

We recovered one female progeny with white eyes and cut wings out of 2,500 females screened. To fully characterize the new balancer, we performed whole genome paired-end DNA sequencing (SRX17766708). When we mapped these reads to the reference genome, we found 5 discordant read pairs between *w* and *ct* spanning the intended gRNA cut sites, and *vice versa,* indicating that these regions had been brought into proximity by a new inversion ( *In(1)FM8)* . PCR confirmed these breakpoints and Sanger sequencing amplicons indicated that the segments were joined at position [chrX:7,535,590|chrX:2,795,833] and position [chrX:7,535,595|chrX:2,795,820] along the X chromosome. The chromosomal order of this novel inversion balancer, which we have named *First Multiple 8* ( *FM8* ), is 1Lt-1B2 | 20F-20A | 15D-20A | 15D-11F4 | 4E1-7B4 | 3B6-4D7 | 11F2-7B4 | 3B6-1B3 | 1Rt, (Figure 1C). *FM8* is a non-viable X chromosome balancer with new mutations that fail to complement *w* and *ct ^6^* , and should further suppress double crossover events that have been shown to occur within the 4E1-11F2 region found in *FM7c* . Unlike *FM8, FM7c* is hemizygous viable, and while we were interested in the lethal chromosome, it should be possible to create a viable variant by inverting within a large intron of a non-essential gene within 4E1-11F2.

The success of this methodology on the X chromosome suggests *Drosophila melanogaster* balancer chromosomes can be created at will. There is clear need for such improvements. For example, the third chromosome balancer *Third Multiple 3* ( *TM3)* has a problematic non-inverted segment on 3L where single and double crossover events occur (Miller et al. 2016b). With this new method, researchers working on genes in such poorly balanced regions can quickly improve existing balancer crossover suppression fidelity. There are many existing and emerging model organisms, including many species of *Drosophila,* where balancer chromosomes do not exist or are difficult to create using transgenes, recombinases, and target sequences (Stern 2022). Our methodology might be applicable to those species, where they would greatly aid in the design of genetic crosses and stock keeping.

## Methods

*U63-gRNA ^w,ct^* contains two gRNAs targeting *w* and two gRNAs targeting *ct* that were separated by tRNAs and was synthesized by GenScript (Piscataway, NJ) and cloned into PCFD5 (Port and Bullock 2016; Benner and Oliver 2018). This new PCFD5- *U63-gRNA ^w,ct^* was integrated into *y ^1^ v ^1^ ; P{y ^+t7.7^ =CaryP}attP40* landing site (Markstein et al. 2008) through phi-C31 integration by *P{nos-phiC31\int.NLS}* (Bischof et al. 2007). Embryo injections for transgenesis and transformant recovery was completed by BestGene (Chino Hills, CA). Genomic DNA was extracted from *FM8* whole flies using the Qiagen DNeasy Blood and Tissue Kit (Hilden, Germany) using the manufacturers insect protocol. 250 uL of 20 ng/uL genomic DNA in TE buffer with 40 mg of Sigma G1277 beads was sonicated for 10 cycles using a Diagenode Bioruptor Plus sonication device (Denville, NJ). For the creation of DNA sequencing libraries, 5 ug of input DNA was used in the NEBNext Ultra II DNA Library Prep Kit for Illumina (Ipswich, MA) according to its standard protocol. The libraries were sequenced using the NovaSeq Xp work flow cell on the NovaSeq 6000 System (San Diego, CA). Raw sequencing reads are available through the SRA (SRX17766708). Sanger sequencing was completed by Genewiz (Plainfield, NJ). Flies were raised on ‘Fly Food B’ (LabExpress, Ann Arbor, MI) under standard conditions at 25°C.

## Reagents

*P{nos-phiC31\int.NLS}* (FBal0193775)

*y ^1^ v ^1^ ; P{y ^+t7.7^ v ^+1.8^ =U6:3-gRNA ^w,ct^ }attP40/CyO* (This study)

*y ^1^ v ^1^ ; P{y ^+t7.7^ =CaryP}attP40* (FBst0036304)

*y ^1^ w ^1^ ct ^6^* ( *y ^1^* = FBal0018607, *w ^1^* = FBal0018074, *ct ^6^* = FBal0001934)

y ^1^ sc ^*^ v ^1^ sev ^21^ ; P{y ^+t7.7^ v ^+1.8^ =nos-Cas9.R}attp40 (FBst0078781)

*In(1)FM7, y ^31d^ sc ^8^ w ^a^* *v ^Of^ g ^4^ B ^1^* (FBab0003929)

*In(1)FM8* , *y ^31d^ sc ^8^ w ^FM8^ ct ^FM8^ v ^Of^ g ^4^ B ^1^* (This study)

**Primer Sequences**

w-ct-inv-F: TCCCGTTTTGTCGATTGTGC

w-ct-inv-R: CCGCAAAGGGTCCATTACCA

ct-w-inv-F: TCGACTCGCAGATTGGTAGC

ct-w-inv-R: TGCCAAAACTCCTCTCGCTT

**gRNA Sequences**

*w* gRNA 1: TAGGGACGCATAACCAGTGG

*w* gRNA 2: ACGCATAACCAGTGGTGGCG

*ct* gRNA 1: TGACAGGCCCGCAAATCGCT

*ct* gRNA 2: CGATCAATCAGCGATTTGAC

## References

[R1] Benner L, Oliver B (2018). Drosophila balancer reengineering using polycistronic gRNA for CRISPR/Cas9 gene editing.. MicroPubl Biol.

[R2] Bischof J, Maeda RK, Hediger M, Karch F, Basler K (2007). An optimized transgenesis system for Drosophila using germ-line-specific phiC31 integrases.. Proc Natl Acad Sci U S A.

[R3] Gramates LS, Agapite J, Attrill H, Calvi BR, Crosby MA, Dos Santos G, Goodman JL, Goutte-Gattat D, Jenkins VK, Kaufman T, Larkin A, Matthews BB, Millburn G, Strelets VB, the FlyBase Consortium. (2022). Fly Base: a guided tour of highlighted features.. Genetics.

[R4] Jack JW (1985). Molecular organization of the cut locus of Drosophila melanogaster.. Cell.

[R5] Markstein M, Pitsouli C, Villalta C, Celniker SE, Perrimon N (2008). Exploiting position effects and the gypsy retrovirus insulator to engineer precisely expressed transgenes.. Nat Genet.

[R6] Merriam JR. FM7: First multiple seven. *Dros Inf Serv. * 1968;43:64

[R7] Miller DE, Cook KR, Yeganeh Kazemi N, Smith CB, Cockrell AJ, Hawley RS, Bergman CM (2016). Rare recombination events generate sequence diversity among balancer chromosomes in Drosophila melanogaster.. Proc Natl Acad Sci U S A.

[R8] Miller DE, Cook KR, Arvanitakis AV, Hawley RS (2016). Third Chromosome Balancer Inversions Disrupt Protein-Coding Genes and Influence Distal Recombination Events in Drosophila melanogaster.. G3 (Bethesda).

[R9] Miller DE, Cook KR, Hawley RS (2019). The joy of balancers.. PLoS Genet.

[R10] Miller DE, Kahsai L, Buddika K, Dixon MJ, Kim BY, Calvi BR, Sokol NS, Hawley RS, Cook KR (2020). Identification and Characterization of Breakpoints and Mutations on
*Drosophila melanogaster*
Balancer Chromosomes.. G3 (Bethesda).

[R11] Muller HJ (1918). Genetic Variability, Twin Hybrids and Constant Hybrids, in a Case of Balanced Lethal Factors.. Genetics.

[R12] Port F, Bullock SL (2016). Augmenting CRISPR applications in Drosophila with tRNA-flanked sgRNAs.. Nat Methods.

[R13] Ren X, Sun J, Housden BE, Hu Y, Roesel C, Lin S, Liu LP, Yang Z, Mao D, Sun L, Wu Q, Ji JY, Xi J, Mohr SE, Xu J, Perrimon N, Ni JQ (2013). Optimized gene editing technology for Drosophila melanogaster using germ line-specific Cas9.. Proc Natl Acad Sci U S A.

[R14] Stern DL (2022). Transgenic tools for targeted chromosome rearrangements allow construction of balancer chromosomes in non-melanogaster Drosophila species.. G3 (Bethesda).

